# Serum ‘Vitamin-Mineral’ Profiles: Associations with Postmenopausal Breast Cancer Risk Including Dietary Patterns and Supplementation. A Case-Control Study

**DOI:** 10.3390/nu11092244

**Published:** 2019-09-18

**Authors:** Beata Krusinska, Lidia Wadolowska, Maciej Biernacki, Malgorzata Anna Slowinska, Marek Drozdowski

**Affiliations:** 1 Department of Human Nutrition, University of Warmia and Mazury in Olsztyn, Sloneczna 45f, 10-718 Olsztyn, Poland; lidia.wadolowska@uwm.edu.pl (L.W.); malgorzata.slowinska@uwm.edu.pl (M.A.S.); 2 Department of Surgery, University of Warmia and Mazury in Olsztyn, 11-041 Olsztyn, Poland; maciej.biernacki@uwm.edu.pl; 3 Department of Laboratory Medicine, University of Warmia and Mazury in Olsztyn, 11-041 Olsztyn, Poland; marek.drozdowski@uwm.edu.pl

**Keywords:** breast cancer, serum vitamin level, serum mineral level, dietary pattern, supplementation

## Abstract

Breast cancer is the most prevalent cancer in females worldwide. Studies evaluating the blood vitamins and minerals status in the breast cancer etiology are limited, and the results are inconclusive. This study analyzed the association between serum vitamin-mineral profiles (V-MPs) and breast cancer (BC) risk with including dietary patterns (DPs) and the use of supplements. This case-control study involved 420 women aged 40–79 years from north-eastern Poland, including 190 newly diagnosed breast cancer cases. The fasting serum concentrations of vitamins (folate, cobalamin, 25(OH) vitamin D) and minerals (iron, calcium, magnesium) were measured in 129 post-menopausal women, including 82 controls and 47 cases. Three V-MPs were derived with a Principal Component Analysis (PCA). A logistic regression analysis was performed to estimate the odds ratio (OR) and 95% confidence interval (95% CI) of the breast cancer risk associated with serum V-MPs and serum levels of single biomarkers. The risk of BC was lower by 88% (OR: 0.12; 95% Cl: 0.02–0.88; *p* < 0.05) in the upper tertile of the serum ‘Iron-Calcium’ profile compared to the bottom tertile, lower by 67% (OR: 0.33; 95% Cl: 0.11–0.97; *p* < 0.05) at the level of serum 25(OH) vitamin D ≥24.6 ng/mL and lower by 68% (OR: 0.32; 95% Cl: 0.11–0.91; *p* < 0.05) at the level of serum calcium ≥9.6 mg/dL. There was an inverse association of the serum ‘Magnesium’ profile or serum level of iron with the risk of BC, which disappeared after adjustment for the set of confounders accounted for: age, body mass index (BMI), socioeconomic status, overall physical activity, smoking status, age at menarche, number of full-term pregnancies, oral contraceptive use, hormone-replacement therapy use, family history of breast cancer, vitamin/mineral supplement use, the molecular subtype of breast cancer, and dietary patterns. No significant association was found between BC risk and the serum ‘Folate-Cobalamin-Vitamin D’ profile or serum folate, cobalamin or magnesium considered separately. These findings highlight that a higher-normal serum level of both iron and calcium, considered together as the serum profile, as well as a higher-normal serum level of calcium, considered separately, and a slightly below the normal range of serum vitamin D level may protect against breast cancer among postmenopausal women, independent of dietary patterns or the use of vitamin/mineral supplements. Therefore, the maintenance of the adequate status of vitamins and minerals and the regular monitoring of their blood markers should be included in breast cancer prevention.

## 1. Introduction

Breast cancer is the most commonly diagnosed cancer and the leading cause of cancer deaths among women worldwide [1]. It is considered an important public health problem throughout the world, especially in economically developed and developing countries [1]. It accounts for approximately 24% of the total female cancer cases and 15% of the total female cancer deaths worldwide [2]. In Poland, breast cancer is the most commonly diagnosed cancer and is second only to lung cancer of cancer deaths in women [3]. Breast cancer accounts for approximately 22% and 14% of total female cancer cases and deaths in Polish females, respectively [1]. The risk of breast cancer increases with age. It is estimated that as many as 80% of breast cancer incidences occur in postmenopausal women, with nearly 50% of cases diagnosed between 50–69 years of age [3].

Besides age, the multifactorial etiology of breast cancer also includes: Genetic, reproductive, and lifestyle factors [4]. A direct cause of carcinogenesis are mutations in genes encoding proteins involved in the cell cycle [5]. Gene mutations and aberrant gene expression could result from epigenetic mechanisms, including aberrations of DNA (deoxyribonucleic acid) methylation, which can be modified by environmental factors such as diet [6]. The involvement of nutrients in cancer etiology include direct mechanisms through interactions with genes or indirect actions through an influence on DNA [6]. In the context of breast cancer risk, the current findings are mostly focused on single nutrient intake, with limited evidence of blood biomarker levels, which may better reflect nutrient status from various sources, including supplements and endogenous synthesis, not only dietary sources [4,7,8,9,10].

In regard to the water-soluble B-vitamins, folate, and vitamin B12 are important co-factors involved in one-carbon metabolism which plays a role in DNA synthesis, methylation, stability, and repair [8]. There are limited studies evaluating the association between serum folate or vitamin B12 and human breast cancer risk, and the results are mixed [11]. Some large prospective and case-control studies suggest an inverse association between folate and vitamin B12 status and breast cancer [11,12], while other cohort studies suggest that high levels of these vitamins may increase the risk of cancer [9,13,14,15]. This inconsistency among various studies may be related to differences in study design, sample sizes, statistical methods, the set of confounders taken into account, and the use of different cut-off points according to serum levels of these vitamins. Regarding fat-soluble vitamins, many beneficial roles in mammary cells, through immune-related functions and DNA methylation regulation, are played by vitamin D [16]. The anticarcinogenic properties of vitamin D were found in cellular in vitro models [17]. A protective effect of vitamin D against human breast cancer has been reported in most of the observational studies [18,19,20,21,22,23,24,25,26,27,28,29], although not all [30,31,32].

In regard to the minerals, calcium (Ca), magnesium (Mg), and iron (Fe) also appear to be involved in breast cancer etiology [33,34]. Calcium is an important intracellular cation, which plays a role in cell signaling, regulating cell proliferation, differentiation, and apoptosis [33]. Decreased intracellular calcium level may stimulate the proliferation of cancer-transformed cells with simultaneous inhibition of cell differentiation and apoptosis, which promotes tumor growth [33]. There is limited data regarding the association between serum calcium levels and the risk of breast cancer among women and the findings are inconsistent [31,35,36,37,38,39]. This inconsistency could result from methodological differences between the studies, particularly the menopausal status of the enrolled sample, which associated calcium metabolism and breast density [36]. Magnesium is essential for DNA duplication and repair, and Mg deficiency promotes DNA mutations leading to carcinogenesis [38]. There is a lack of data on the serum magnesium level associated with the risk of breast cancer. Recently, it has been suggested that the ratio of serum calcium to magnesium may be linked with the risk of cancer [38]. Iron is an essential micronutrient which plays an important role in oxygen transport and DNA synthesis. On the other hand, iron catalyzes the production of reactive oxygen species, resulting in increased oxidative stress, mutations, DNA breaks, and oncogenic activation [39]. The carcinogenic potential of iron shown in the in vitro models [39] was supported by the results from observational studies related to iron status and postmenopausal breast cancer incidence [40,41,42,43]. However, there is limited data in this area. 

B-group vitamins, vitamin D, calcium, magnesium, and iron play a role at various stages of cancer development; from an impact on cellular genetic material and gene expression to a role in DNA synthesis, methylation, and stability, and an influence on cell proliferation, differentiation, and apoptosis [6,17,33,38,39]. The available studies have examined the association of single blood vitamin and mineral level with human breast cancer [12,14,18,19,20,21,22,23,24,25,26,27,28,29,32,33,35,36,37,39,40,41,42]. This approach does not include the interactions between the various biomarkers. The metabolism of nutrients is interrelated. An example would be the interaction of B-group vitamins, calcium, and vitamin D and calcium, magnesium, and iron [11,34,36,38]. Therefore, nutrient-nutrient interactions could impact cell metabolism and result in the weakening or strengthening of the risk of cancer [44]. Given the complex etiology of breast cancer, possible nutrient-nutrient interactions and the common mechanisms involving the mentioned nutrients in cancer etiology proven in vitro model studies, a comprehensive approach focused on both vitamins and mineral serum concentrations is needed to provide clear evidence of nutrient-breast cancer associations in observational human studies.

The aims of the study were: (1) to assess the breast cancer risk for a higher versus lower level of serum folate, vitamin B12, vitamin D, iron, calcium, and magnesium; and (2) to evaluate the joint association of these biomarkers as serum vitamin-mineral profiles (V-MPs) with the risk of breast cancer in postmenopausal women from north-eastern Poland. In these observations, many potential confounders, including dietary patterns were taken into account. Dietary patterns (DPs) express the complexity of the diet and are currently considered the best approach to evaluating the diet as a matrix of various foods and therefore the main source of nutrients status [4]. Thus, it was decided to take into account DPs identified based on previous data from the study regarding the association between DPs and breast cancer risk among peri and postmenopausal women from the same region [45]. Furthermore, the self-declared vitamin/mineral supplements use as the additional, next to diet, nutrients sources were also taken into account [10].

## 2. Materials and Methods

### 2.1. Ethical Approval 

This study was approved by the Bioethics Committee of the Faculty of Medical Sciences, University of Warmia and Mazury in Olsztyn on 2 October 2013 (resolution no. 29/2013).

The aim of the study, study stages, and sample collection procedures were explained to all subjects. All subjects gave their written informed consent to participate in the study, including for blood sample collection and to use clinical data for research.

### 2.2. Study Design

**Figure 1:**
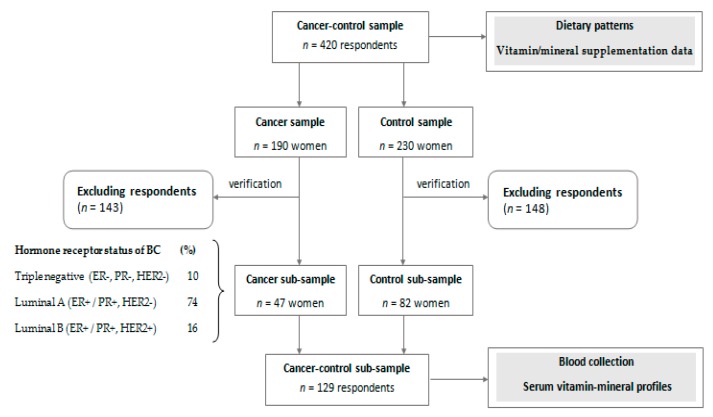
Study design and sample collection. BC—breast cancer; ER—estrogen receptor status of tumor; PR—progesterone receptor status of tumor; HER2—human epidermal growth factor receptor 2.

The present study was a part of a larger case-control study conducted in 2014–2017, concerning dietary patterns and breast cancer risk among 420 women from north-eastern Poland [41]. Briefly, in this study, the cancer-control sub-sample involved 129 subjects, aged 45.0–79.9 (mean 61.9 SD 8.2) years, including 47 newly diagnosed breast cancer cases (cancer sub-sample) and 82 women without any breast pathology based on the mammography and/or breast ultrasonography screening (control sub-sample). All breast cancer cases were diagnosed by oncologists based on histopathology results from a biopsy at the Ministry of Internal Affairs Hospital with the Warmia and Mazury Oncology Centre in Olsztyn. The time from cancer diagnosis to case recruitment in the study ranged from seven to 28 days [45]. Cancer and control subjects were non-randomly recruited from January 2016 to June 2017. Details of the study design and sample collection were described previously [45]. In the cancer cases, the most frequency diagnosed were tumors with positive estrogen (ER+) and/or progesterone receptor status (PR+) and negative human epidermal growth factor receptor 2 (HER2-) of cancer (74%; Luminal A; Figure 1), which are the most common molecular subtype of breast cancers worldwide, including Poland [1]. The inclusion and exclusion criteria of the cancer and control sub-samples collection are shown in Table 1. All data were collected before treatment or surgical intervention in cancer cases to avoid reverse causation.

### 2.3. Blood Samples and Laboratory Analyses

Fasting venous blood samples were collected for 129 postmenopausal women, including 47 preoperative breast cancer cases and 82 controls. The procedure of the blood sample collection and the serum obtaining were described previously [45]. In brief, a 12 mL blood samples were taken in two 6 mL red top tubes (Clot Activator Tube; BD Vacutainer^®^, Franklin Lakes, NJ, USA). Blood samples stood for 30 min at 22 °C, and then were centrifuged at 3000 rpm for 10 min at 22 °C, which allowed the separated serum obtained. The case-control status of the blood samples was blinded. All methods were fully automated and performed at the Laboratory of Biochemical Studies of Nutritional Status in the Department of Human Nutrition at the University of Warmia and Mazury in Olsztyn.

Iron, calcium, and magnesium concentrations were measured in serum samples using a Cobas Integra 400 plus auto-analyzer (Roche Diagnostics^®^, Basel, Switzerland) according to the manufacturer’s instructions. Serum concentrations of folate, cobalamin, and 25(OH) vitamin D were measured with electrochemiluminescence immunoassays (ECLIA) using an automated immune-analyzer Cobas e411 (Roche Diagnostics^®^, Basel, Switzerland). The minimum detectable concentrations (MDC) were as follows: iron 5.00 μg/dL, calcium 0.8 mg/dL, magnesium 0.36 mg/dL, folate 0.640 ng/mL, cobalamin 100 pg/mL, and 25(OH) vitamin D 3.00 ng/mL.

### 2.4. Identification of Serum Vitamin-Mineral Profiles

The Principal Component Analysis (PCA) with varimax rotation was used to identify (a posteriori approach) serum vitamin-mineral profiles (V-MPs). The values of serum vitamins and mineral concentrations were standardized and then included into the PCA. During identification of the number of serum V-MPs, the following criteria were considered: (i) the eigenvalues of the variable correlations >1.0, (ii) the plot of eigenvalues, and (iii) the total variance explained [46]. Serum V-MPs were identified according to their main components with absolute factor loadings >|0.50|, which indicated a strong association between biomarker and the serum V-MP.

Three serum V-MPs were identified (Table 2). The ‘Folate-Cobalamin-Vitamin D’ profile was positively loaded by the serum concentration of folate, cobalamin and 25(OH) vitamin D. The ‘Iron-Calcium’ profile was positively loaded by the serum concentration of iron and calcium. The ‘Magnesium’ profile was positively loaded by the serum concentration of magnesium (Table 2). For each of the serum V-MPs, tertile intervals, as well as, the V-MP scores (in points) as a sum of the product of the biomarkers values and its factor loadings were calculated.

### 2.5. Dietary Assessment

Dietary data were collected for 420 peri and postmenopausal women, including 190 breast cancer cases and 230 controls [45]. The frequency consumption of 62 food groups at least 12 months prior to participation in the study was obtained using a validated the 62-item Food Frequency Questionnaire (FFQ-6) [47,48]. The frequency consumption of some food groups (expressed as times/day) was summed up to form 21 food groups (Table S1), and then was standardized and input into the PCA with varimax rotation to identify dietary patterns (Table 3). The criterions of the PCA-derived DPs identification were described previously [45]. For each of the DPs, the DPs scores (in points) as a sum of the product of the frequency of food consumption and its factor loadings were calculated.

### 2.6. Confounders

Data regarding socioeconomic, lifestyle, reproductive, and medical factors were collected in an individual interview with each participant. The set of confounders included the factors given below, which can be involved in the breast cancer etiology [4]:

- age;
- body mass index;
- socioeconomic status;
- overall physical activity;
- smoking status;
- age at menarche;
- number of full-term pregnancies;
- oral contraceptive use;
- hormone-replacement therapy use;
- family history of breast cancer;
- vitamin/mineral supplement use;
- molecular subtype of breast cancer;
- PCA-derived dietary patterns (in scores).

Respondents were asked about four single factors to describe their socioeconomic status (SES). The SES index was calculated as the sum of the values assigned to the response categories of each SES factor (Table 4). The SES index values were logarithmized and the tertiles of the SES were then created to identify respondents with low, average, and high SES.

Respondents were asked to describe their physical activity at work and physical activity in leisure time by choosing one of three categories (Table 5). Self-declared data was then combined, and three categories of overall physical activity were created: low, moderate, and high (Table 6).

### 2.7. Statistical Analysis

The serum biomarker concentrations were expressed by the mean and standard deviation (SD). The continuous log values of biomarkers and Student’s t-test were used to compare them between cases and controls. The median (Me) of the biomarker concentrations was used to divide subjects into two categories (<Me, ≥Me). The categorical variables were presented in percentages. Differences in baseline case-control characteristics were verified with a Pearson Chi^2^ test (categorical data) or a Kruskal-Wallis test (continuous data) [46]. The percentage distribution of breast cancer cases was compared by tertiles of serum V-MPs using Pearson Chi^2^ test with Yates’ correction as necessary. Logistic regression analysis was used to estimate the odds ratio (OR) and 95% confidence interval (95% CI) of the breast cancer risk in associations with serum V-MPs and serum levels of single biomarkers. The references (OR = 1.00) were the control sample and the bottom tertile of each serum V-MPs or lower level of single biomarker concentration (<Me). Three models were created: model 1—unadjusted, model 2—adjusted for the aforementioned set of confounders, and model 3—fully-adjusted for the same confounders included in the model 2 and for DP scores. The level of significance of the OR was verified with Wald’s test [46]. *p* values <0.05 were considered as statistically significant. All data were analyzed using STATISTICA software (version 13.0 PL; StatSoft Inc., Tulsa, OK, USA; StatSoft, Krakow, Poland). 

## 3. Results 

### 3.1. Baseline Sample Characteristics

The baseline characteristics of the cancer-control sub-sample are shown in Table 7. All women were postmenopausal. Cancer cases and controls did not differ in age or BMI. Compared to the controls, more breast cancer cases occurred among women with lower socioeconomic status, more were less physically active or had at least three full-term pregnancies. Breast cancer cases had, on average, a higher score of the ‘Non-healthy’ dietary pattern than controls. About half of the participants self-declared use of vitamin and/or mineral supplements within the last 12 months. None of the participants abused alcohol (Table 7). The comparative characteristics of cancer and control samples with their sub-samples are shown in Supplementary Tables S2–S4. 

### 3.2. Vitamin-Mineral Profiles and Breast Cancer Risk

Breast cancer cases had a lower mean score of the ‘Folate-Cobalamin-Vitamin D’ profile (300.8 versus 349.3 points), and the ‘Iron-Calcium’ profile (127.0 versus 142.7 points) than controls. Compared to the controls, the number of breast cancer cases was lower in the upper tertile of the ‘Iron-Calcium’ profile (24.5 versus 39.5%), and the ‘Magnesium’ profile (18.4 versus 43.2%) than the bottom tertile (Table 8).

Compared to the controls, breast cancer cases had a lower mean serum concentration of most of the selected vitamins and minerals: 25(OH) vitamin D (21.9 versus 29.5 ng/mL), iron (97.8 versus 108.3 µg/dL), calcium (9.5 versus 9.6 mg/dL), and magnesium (2.0 versus 2.1 mg/dL), excluding folate and cobalamin. Compared to the controls, fewer cases of breast cancer had elevated levels of serum concentration of 25(OH) vitamin D ≥24.6 ng/mL (34.0 versus 60.5%), iron ≥103.0 µg/dL (38.0 versus 58.0%), and calcium ≥9.6 mg/dL (46.0 versus 69.1%; Table 8). On the other hand, more cancer cases had elevated levels of serum folate concentration ≥10.5 ng/mL (61.2 versus 43.2%) than the controls. The mean ratio of serum calcium to magnesium was 4.6 for the total sample and was not significantly different between cases and controls (Table 8). The histograms of serum 25(OH) vitamin D and calcium concentrations among cancer and control sub-samples are shown in Figure S1. The sub-sample characteristics and serum concentration of vitamins and minerals by tertiles of serum V-MPs are shown in Supplementary Tables S5 and S6, respectively.

In the upper tertile of the serum ‘Iron-Calcium’ profile, the risk of breast cancer was lower by 66% (OR: 0.34; 95% Cl: 0.14–0.84; *p* < 0.05; unadjusted model 1; reference: bottom tertile) and 88% (OR: 0.12; 95% Cl: 0.02–0.88; *p* < 0.05; fully-adjusted model 3; reference: bottom tertile; Table 9). In the upper tertile of the serum ‘Magnesium’ profile, the risk of breast cancer was lower by 77% (OR: 0.23; 95% Cl: 0.09–0.61; *p* < 0.01; unadjusted model 1; reference: bottom tertile) and 79% (OR: 0.21; 95% Cl: 0.05–0.84; *p* < 0.05; adjusted model 2; reference: bottom tertile). This association disappeared after the adjustment for dietary patterns (fully-adjusted model 3). The serum ‘Folate-Cobalamin-Vitamin D’ profile was not significantly associated with the risk of breast cancer (Table 9).

In the higher level of the serum vitamin D concentration (≥24.6 ng/mL), the risk of breast cancer was lower by 67% (OR: 0.33; 95% Cl: 0.11–0.97; *p* < 0.05; fully-adjusted model 3) and 72% (OR: 0.28; 95% Cl: 0.10–0.78; *p* < 0.05; adjusted model 2) compared to the lower level (Table 9). In the higher level of the serum calcium concentration (≥9.6 mg/dL), the risk of breast cancer was lower by 62% (OR: 0.38; 95% Cl: 0.18–0.79; *p* < 0.01; unadjusted model 1) and 68% (OR: 0.32; 95% Cl: 0.11–0.91; *p* < 0.05; fully-adjusted model 3) compared to the lower level. In the higher level of the serum iron concentration (≥103.0 μg/dL), the risk of breast cancer was lower by 56% (OR: 0.44; 95% Cl: 0.21–0.92; *p* < 0.05; unadjusted model 1) compared to the lower level. This association disappeared after the adjustment. There were no significant associations of the serum level of folate, cobalamin, and magnesium considered separately with the risk of breast cancer (Table 9). 

## 4. Discussion

To the authors’ best knowledge, this is the first study describing the association between serum vitamin-mineral profile and breast cancer risk, which takes into account dietary patterns and vitamin/mineral supplement use in this area. The findings highlight the clear protective effect of the serum ‘Iron-Calcium’ profile as well as the protective effect of single biomarkers such as the higher-normal serum level of calcium and the slightly below the normal range of serum vitamin D level on the risk of breast cancer in postmenopausal women from north-eastern Poland. An inverse association of the serum ‘Magnesium’ profile and serum iron level with the risk of cancer was found, but it disappeared after adjustment. There was no significant association between serum ‘Folate-Cobalamin-Vitamin D’ profile or serum level of folate and cobalamin considered separately and cancer.

### 4.1. Serum Vitamins and Minerals and Breast Cancer Risk 

The findings provide new insight into the importance of serum vitamins and minerals in breast cancer prevention. The ‘Iron-Calcium’ profile was positively loaded by the serum concentration of these minerals. A high adherence to the serum ‘Iron-Calcium’ profile was associated with an 88% decrease in breast cancer risk, and remained significant after adjustment for many confounders, including dietary patterns and supplement use. This strong association was obtained in a case-control study with a smaller strength of inference and needs to be confirmed in large prospective studies. Since the available studies have been focused mainly on single biomarkers in association with breast cancer, it is hard to compare the current results with others. In regard to the single serum minerals, it was found that a higher serum level of calcium (≥9.6 mg/dL) within the normal laboratory range (from 8.5 to 10.5 mg/dL) [51] was associated with a 68% lower risk of breast cancer. These results are consistent with the findings from the Swedish Apolipoprotein Mortality Risk (AMORIS) prospective Study, which included 229,674 women and showed an inverse association between serum calcium and the risk of breast cancer, although this association was weaker [35]. A higher level of serum calcium (≥9.8 mg/dL) reduced the risk of breast cancer by 6% [35]. The protective role of calcium on breast tissue could possibly result from the influence on reduced breast density as an established risk factor of breast cancer [36]. This beneficial role of calcium was observed in the upper range of values considered normal, not above, because calcium homeostasis is carefully regulated by many mechanisms to maintain constant serum concentration [33]. In contrast, in the nested case-control Malmo Diet Cancer Study including 764 cases and 764 controls, serum calcium was positively associated with breast cancer, but only in premenopausal women [31]. However, in a prospective cohort study of 2338 women, Spraque et al. [37] showed no association between serum calcium and breast cancer risk was found, either overall or stratified by menopausal status.

Calcium metabolism is closely related to the vitamin D status [36]. Similar to the current results, in the French cohort study which obtained 1,908 women [18] was found that a 25(OH) vitamin D serum concentration was lower in cases compared to the controls (21.9 versus 29.5 ng/mL, and 24.4 versus 25.1 ng/mL, respectively), and this association was reported in other studies [19,20]. The serum level of 25(OH) vitamin D (≥24.6 ng/mL) was slightly below the normal range (>30 ng/mL) [52] and was associated with a lower risk of breast cancer by 67%. This inverse association was also reported in most of the available studies [18,19,20,21,22,23,25,26,27,28,29,53]. The reduction in postmenopausal breast cancer risk ranged from 30% during the five year follow-up Sister Cohort Study of 3386 women [23] to 65% in the pooled analysis of the randomized trial and prospective cohort study covering a total of 2304 women [22], when the serum 25(OH)D was more than 38.0 ng/mL and 40 ng/mL, respectively. Such protective effect of vitamin D could be explained by regulation of cell proliferation and immune function, inhibition of metastasis and angiogenesis as well as induction of cell differentiation and apoptosis [54]. In addition, vitamin D inhibits the insulin-like growth factor I (IGF-I), which stimulates the growth of breast cancer cells [16]. However, other studies found no significant association between vitamin D levels and breast cancer risk [31,32]. Unexpectedly, the meta-analysis of three large European population-based cohort studies, conducted by Ordóñez-Mena et al. [30], showed increased breast cancer risk with higher 25(OH)D concentrations. This inconsistency among different studies may be related to differences in sample sizes, enrolled populations, study design, season of blood donation, and vitamin D measurements, the set of confounders taken into account and use different cut-off points according to serum levels of vitamin D were considered. Moreover, Shirazi et al. [27] in the nested case-control Malmo Diet and Cancer Study reported that women in the first and third tertile of the serum 25OH D3 level had a similar risk of breast cancer. Thus, these results suggested that both significant deficiencies and excessive levels of vitamin D are unfavorable.

Although high adherence to the serum ‘Magnesium’ profile decreased the risk of breast cancer, this association disappeared in the fully-adjusted model. There is a lack of epidemiological or clinical studies regarding the association between serum magnesium level and breast cancer risk. The molecular mechanism of the magnesium role in breast cancer etiology can be explained by a recent experimental study which suggested that the over-expression of oncogenic protein tyrosine phosphatase PRL-2 can promote oncogenesis by modulating intracellular magnesium levels through binding with the magnesium transporter [55]. The available data concerning the serum calcium to magnesium ratio in relation to breast cancer risk [38]. In the present study, the mean ratio of serum Ca/Mg was 4.6 and was not significantly different depending on the cancer. In contrast, Sahmoun and Singh [38] showed that the serum Ca/Mg ratio was higher in breast cancer cases than controls (4.9 versus 4.4), suggesting that a high serum Ca/Mg ratio may be a risk factor for postmenopausal breast cancer. However, these results were obtained from the small preliminary data from medical charts review [38] and require confirmation in large studies. Nevertheless, an imbalance of the serum Ca/Mg can lead to irregularities in mutually modulated signaling pathways and then to increase the risk of incorrect DNA repair, cell proliferation, and, consequently, to carcinogenesis [38].

Identification of the serum ‘Iron-Calcium’ profile supports the positive correlation between calcium and iron shown by Joo et al. [34] in a study with breast cancer patients. In the present study, the beneficial effect of the higher serum level of iron as a single biomarker was weak and disappeared after adjustment. However, a significant reduction in the risk of breast cancer was found with the elevated ferritin levels as one of the iron status markers in the prospective population-based EPIC-Heidelberg Study covering 627 breast cancer cases [40]. Conversely, in a Swedish cohort study [42] and in the prospective Busselton Health Study [43] involving 108,823 and 1795 women, respectively, elevated serum iron levels were associated with a greater risk of postmenopausal breast cancer. Contrary to the current results, Bae et al. [41] found a higher mean serum iron concentration and elevated serum iron level in breast cancer cases than in the controls, with 121 and 149 subjects, respectively. Excess iron may induce oxidative stress through the production of reactive oxygen species and lead to oxidative DNA damage and mammary carcinogenesis [41]. Furthermore, it was suggested that the higher incidence of breast cancer in postmenopausal women may result from two to three times higher serum Fe status than in premenopausal women due to cessation of menstrual bleeding [41].

Likewise, in some other studies, including the recent meta-analysis of four studies, no significant associations between the serum level of folate [56,57] or vitamin B12 [11,13] were found with the risk of breast cancer. However, Rossi et al. [12] in the prospective study conducted in the cohort of 1024 women, reported an inverse association between red blood cell folate levels and breast cancer risk mortality and Wu et al. [11] found a weak inverse association between serum vitamin B12 and breast cancer risk for the seven case-control studies that were separately considered. Several biological mechanisms for these inverse associations have been proposed. A deficiency in folate or vitamin B12 may result in an increase in genetic aberrations which have been implicated in neoplastic transformation [8]. Moreover, low B-group vitamins concentrations are associated with inflammation and higher oxidative stress which may lead to the carcinogenesis [8]. Interestingly, a positive association with the risk of breast cancer was observed for the high levels of vitamin B12 in women who abused alcohol [9] and for the high folate levels in women overall [13] as well as in women with BRCA1/2 mutations [15] and in women with estrogen receptor beta negative tumors [14]. The increased risk was attributed to excess supplementation. The aforementioned studies suggest both very low and very high folate levels could produce the potential harmful effects of folate due to abnormalities in DNA synthesis. 

### 4.2. Strengths and Limitations 

A major strength of this study is the novel approach in the breast cancer risk assessment by considering together blood concentrations of several vitamins and minerals as overall vitamin-mineral profiles. This is the first study to include the combined effects of serum levels of folate, vitamin B12, vitamin D, calcium, iron, and magnesium on breast cancer risk. The strengths of the current study include the serum biomarker measurements, which are better reflected in the circulation nutrient status than other measures of exposure (e.g., dietary intake) and are not influenced by a recall bias [10]. Blood levels are the result of both dietary intake, supplementation, and endogenous synthesis, e.g. as an effect of sun exposure in the case of vitamin D [10]. Moreover, the measured serum 25-hydroxyvitamin D (25OH D) is currently considered the best marker of vitamin D status. Its half-life of about two to three weeks is 1000-fold longer than the half-life of 1,25(OH)2D [21]. Furthermore, all measurements, including the concentration of biomarkers, were made for the newly diagnosed breast cancer cases, prior to treatment or surgery, to avoid reverse causation, and minimize misclassification. Moreover, in order to exclude possible interference affecting vitamins and minerals levels, the blood sampling inclusion criteria were very restrictive and including fasting status, lack of diagnosed chronic diseases, and infections. Finally, in comprehensively evaluating the breast cancer risk, a wide range of potential confounders, including previously identified dietary patterns and respondents’ statements about vitamin/mineral supplement use (or not) as additional nutrient sources were also taken into account. These strengthen the findings of the present study. Nevertheless, there are many other risk factors associated with breast cancer apart from diet and nutrients. The cancer etiology is complex and likely to be related to a combination and interactions of many factors, with included the immunology system condition and environmental pollution which are difficult to measure [5].

There are several study limitations. First, it was a relatively small sub-sample size. The cancer sub-sample size was limited by the number of breast cancer patients of the oncological surgery department in one hospital. Moreover, only newly diagnosed breast cancer cases, not benign or remission, qualified. However, the sample size (129) was greater than the minimum sample size (at least 42 of the controls and 42 of the cases) when calculated using the WHO sample size calculator considering 80% test power and 5% significance level [58]. The second limitation is connected with the study design and sampling bias [59]. A non-random sample selection may reduce the strength and quality of evidence. The associations obtained from case-control studies compared to the prospective studies could be overstated due to recall or selection bias [59]. Nevertheless, after recruited cases and controls at the blood sampling, there was no need for further matching of these groups because of the lack differences in age and BMI between them. Thirdly, while assessing the cancer risk, the type and amount of nutrients taken with supplements, as well as the frequency of supplements use were not considered. In fact, an attempt was made to collect such data, but many respondents were unable to provide the composition of the supplements they took or name them, so a huge amount of data was missing. Therefore, respondents were only classified as either taking vitamin/mineral supplements within the last 12 months or not. Fourthly, a single blood draw for each subject was used for the measurement of biomarker levels and may not be representative in the long-term. However, in a recent cohort NHANES study that measured 25(OH) D at two times, three years apart, found a high inter-correlation between levels [21]. In regard to calcium, both short-time and long-time intra-individual variations in total serum calcium are low [37]. Thus, a single measurement of serum biomarkers is considered a valid marker of the status of selected nutrients. 

### 4.3. Clinical Impact and Future Directions 

The present findings indicate the need to include the monitoring of vitamin and mineral status, particularly calcium and vitamin D, as endogenous markers in both conventional screening of breast cancer and in the treatment protocol. Further prospective studies with longitudinal measurements of serum biomarkers levels as well as randomized clinical trials are needed to establish cut-offs of the levels of vitamins and minerals which help to protect against breast cancer. In a further explanation of the role of nutrients in breast cancer etiology, genetic mutation carriers which have a genetic predisposition to develop highly aggressive cancers should be taken into account [44]. A better understanding of the impact of nutrients on the different stages and phenotypes of cancer development is essential for personalized treatment therapies directed towards nutrient-gene and nutrient-nutrient interactions and with prognostic significance for patient outcomes [44]. This will have important clinical implications.

## 5. Conclusions

In summary, the study revealed that a higher-normal serum level of both iron and calcium, considered together as the serum profile, as well as a higher-normal serum level of calcium, considered separately, and a slightly below the normal range of serum vitamin D level may protect against breast cancer among postmenopausal women, independent of dietary patterns or the use of vitamin/mineral supplements. In the prevention of breast cancer, special attention should be paid to maintaining adequate blood levels of vitamins and minerals and to preventing deficiencies. Thus, the regular monitoring of blood levels of their biomarkers should be included in a breast cancer reducing strategy.

## Figures and Tables

**Table 1:** Criteria of the cancer and control sub-sample collection.

Inclusion Criteria	Exclusion Criteria
women	diabetes
north-eastern Poland (urban and rural areas)	atherosclerosis
post-menopausal status (natural menopause)	infections
mammography and/or ultrasonography of breasts	autoimmune diseases
without chronic diseases or infections	hormonal disorders
fasting status at time of blood sampling	hysterectomy
consent to blood collection	non-fasting status

**Table 2:** Factor loadings for serum vitamins and mineral concentrations in Principal Component Analysis (PCA)-derived profiles among postmenopausal women (*n* = 129).

Biomarkers	Vitamin-Mineral Profiles		
	‘Folate-Cobalamin-Vitamin D’	‘Iron-Calcium’	‘Magnesium’
Folate	**0.82**	−0.06	−0.07
Cobalamin	**0.81**	0.12	0.04
25(OH) vitamin D	**0.49**	0.05	0.39
Iron	0.09	**0.81**	−0.17
Calcium	−0.02	**0.77**	0.22
Magnesium	0.00	0.01	**0.92**
Share in explaining the variance (%)	28	20	17

Bolded values are marked for the main components of serum PCA-derived profiles with absolute factor loadings ≥0.5.

**Table 3:** Description of PCA-derived dietary patterns (DPs) among peri and postmenopausal women (*n* = 420).

Dietary Patterns	Components ^1^ of DPs (Factor Loadings ^2^) Related to Food Consumption Frequency ^3^ of:
‘Non-Healthy’	refined cereals and fine groats (0.67), red and processed meats (0.63), sugar, honey and sweets (0.57), potatoes (0.55), animal fats (0.49), vegetable based oil (0.34), sweetened beverages and energy drinks (0.32), wholemeal cereals and coarse groats (−0.45), nuts and seeds (−0.39);
‘Prudent’	fruits (0.55), fish (0.49), legumes (0.48), milk and milk beverages-natural and cheese curds (0.48), wholemeal cereals and coarse groats (0.47), fruit, vegetable or vegetable-fruit juices (0.45), eggs and egg dishes (0.44), vegetables (0.42), nuts and seeds (0.42), vegetable based oil (0.36), breakfast cereals (0.35), cheese (0.34);
‘Margarine and Sweetened Dairy’	other fats: margarine, mayonnaise and dressings (0.80), milk beverages-sweetened and flavoured cheese curds (0.36), white meat (0.31), breakfast cereals (0.31), animal fats (−0.66).

^1^ description of the full list of food groups was shown in Supplementary Materials (Table S1) [48]; ^2^ values for the main components of PCA-derived dietary patterns with absolute loadings ≥0.3; and ^3^ the frequency consumption was expressed as times/day after assigning the appropriate values as follows: ‘never or almost never’ = 0; ‘once a month or less’ = 0.025; ‘several times a month’ = 0.1; ‘several times a week’ = 0.571; ‘daily’ = 1; ‘several times a day’ = 2 [48].

**Table 4:** Description of the socioeconomic status factors [45].

Socioeconomic Factors	Categories	Scoring
place of residence	village	1
	town <20,000	2
	town 20,000–100,000 inhabitants	3
	city >100,000 inhabitants	4
educational level	primary	1
	secondary	2
	higher	3
economic situation (self-declared)	below average	1
	average	2
	above average	3
situation of household (self-declared)	we live poorly—I don’t have enough resources even for basic needs (food/clothing/housing fees)	1
	we live very thriftily—I have enough resources only for basic needs (food/clothing/housing fees)	2
	we live thriftily—so I have enough resources for everything	3
	we live well—I have enough resources for everything, but I don’t put off savings	4
	we live very well—I have enough resources for everything and I put off savings	5

Scoring—values assigned to the response categories.

**Table 5:** Description of the categories of physical activity at work and at leisure time [45].

Physical Activity	Categories	Description
at work	low	more than 70% of working time spent sedentary or retired
	moderate	50% of working time spent sedentary and 50% of working time spent in an active manner
	high	70% of working time spent in an active manner or physical work related to great exertion
at leisure time	low	sedentary for most of the time, watching TV, reading books, walking 1–2 h/week
	moderate	walking, bike riding, gymnastics, gardening, light physical activity performed 2–3 h/week
	high	bike riding, jogging, gardening, sport activities involving physical exertion performed more than 3 h weekly

**Table 6:** Estimate the overall physical activity after combining data based on self-reported physical activity at work and physical activity in leisure time [49].

		Physical Activity at Work		
		Low	Moderate	High
Physical activity in leisure time	low	low	low	moderate
	moderate	low	moderate	moderate
	high	moderate	moderate	high

**Table 7:** Cancer-control sub-sample characteristics (% or points).

Variable	Cancer-Control Sub-Sample	Cancer Sub-Sample	Control Sub-Sample	*p*-Value
Sample Size	129	47	82	
Age (years ^#^)	61.9 (8.2)	62.2 (10.4)	61.7 (6.7)	0.5724
BMI (kg/m^2 #^)	27.9 (5.1)	28.8 (5.1)	27.3 (5.1)	0.0743
Socioeconomic status (SES Index ^#^)	9.9 (2.3)	8.4 (1.8)	10.8 (2.1)	0.0001
low	40.8	69.4	23.5	
average	35.4	28.6	39.5	0.0001
high	23.8	2.0	37.0	
Overall physical activity ^1^				
low	56.2	71.4	46.9	
moderate	41.5	26.5	50.6	0.0229
high	2.3	2.0	2.5	
Sample Size	129	47	82	
Smoking status (smoker ^2^)	46.2	55.1	40.7	0.1114
Abuse of alcohol ^3^	0.0	0.0	0.0	1.0000
Age at menarche (years)				
<12	7.7	14.3	3.7	
12–14.9	70.0	67.4	71.6	0.0792
≥15	22.3	18.4	24.7	
Menopausal status				
pre-menopausal	0.0	0.0	0.0	1.0000
post-menopausal	100.0	100.0	100.0	
Number of full-term pregnancies				
0	10.8	4.1	14.8	
1–2	62.3	55.1	66.7	0.0084
≥3	26.9	40.8	18.5	
Oral contraceptive use (ever)	23.8	18.4	27.2	0.2542
Hormone-replacement therapy use (ever)	22.3	20.4	23.5	0.6858
Family history of BC ^4^	23.8	32.7	18.5	0.1278
Vitamin/mineral supplements use ^5^	53.8	42.9	60.5	0.0506
Dietary patterns score ^#^ (points)				
‘Non-Healthy’	2.9 (1.7)	3.6 (1.7)	2.5 (1.5)	0.0003
‘Prudent’	3.4 (1.3)	3.1 (1.5)	3.5 (1.1)	0.0607
‘Margarine and Sweetened Dairy’	−0.1 (0.9)	0.1 (1.0)	−0.3 (0.8)	0.0999

BMI—body mass index; BC—breast cancer; SES—socioeconomic status calculated on the basis of place of residence, education level, self-declared economic situation and self-declared household economic status situation (description in the Section 2.6.); ^1^ calculated after combining data based on self-declared physical activity at work and physical activity in leisure time (description in the Section 2.6.) [49]; ^2^ ever-smoker (current and/or former smoker); ^3^ at least one bottle (0.5 L) of beer or two glasses of wine (300 mL), or two drinks (300 mL), or two glasses of vodka (60 mL) per day [50]; ^4^ in first- or second-degree relative; and ^5^ self-declared use of vitamin and/or mineral supplements within the last 12 months; %—sample percentage; ^#^ mean and standard deviation (SD); *p*-value—level of significance verified with chi^2^ test (categorical variables) or Kruskal-Wallis’ test (continuous variables).

**Table 8:** Serum vitamin-mineral profiles and their components in association with breast cancer (% or points).

Variable	Cancer-Control Sub-Sample	Cancer Sub-Sample	Control Sub-Sample	*p*-Value
Sample Size	129	47	82	
‘Folate-Cobalamin-Vitamin D’ profile				
Score ^#^ (points)	331.0 (135.3)	300.8 (119.5)	349.3 (141.6)	0.0424
tertiles				
bottom	33.1	40.8	28.4	
middle	33.8	28.6	37.0	0.3304
upper	33.1	30.6	34.6	
‘Iron-Calcium’ profile				
Score ^#^ (points)	136.8 (33.0)	127.0 (34.7)	142.7 (30.6)	0.0045
tertiles				
bottom	33.8	46.9	25.9	
middle	32.3	28.6	34.6	0.0417
upper	33.8	24.5	39.5	
‘Magnesium’ profile				
Score ^#^ (points)	10.6 (9.8)	8.4 (8.1)	12.0 (10.5)	0.0543
tertiles				
bottom	33.8	46.9	25.9	
middle	32.3	34.7	30.9	0.0079
upper	33.8	18.4	43.2	
Folate (ng/mL) ^#^	11.8 (7.4)	12.4 (6.5)	11.5 (7.9)	0.4840
≥10.5	50.0	61.2	43.2	0.0465
Cobalamin (pg/mL) ^#^	369.2 (160.9)	334.9 (144.4)	389.9 (167.5)	0.0585
≥341.65	50.0	40.8	55.6	0.1034
25(OH) vitamin D (ng/mL) ^#^	26.6 (13.7)	21.9 (10.2)	29.5 (14.7)	0.0019
≥24.6	50.4	34.0	60.5	0.0032
Iron (μg/dL) ^#^	104.3 (29.8)	97.8 (31.6)	108.3 (28.1)	0.0429
≥103.0	50.4	38.0	58.0	0.0260
Calcium (mg/dL) ^#^	9.6 (0.5)	9.5 (0.5)	9.6 (0.4)	0.0290
≥9.6	60.3	46.0	69.1	0.0086
Magnesium (mg/dL) ^#^	2.1 (0.1)	2.0 (0.1)	2.1 (0.1)	0.0009
≥2.1	63.4	56.0	67.9	0.1696
Ratio of serum calcium to magnesium ^#^	4.6 (0.4)	4.7 (0.5)	4.6 (0.3)	0.1222

%—sample percentage; ^#^ mean and standard deviation (SD); *p*-value—level of significance assessed by chi^2^ test (categorical variables) or Kruskal-Wallis’ test (continuous variables) or Student’s *t*-test (for log-transformed serum biomarkers concentration); *p* < 0.05; ns—statistically insignificant.

**Table 9:** Odds ratios (ORs) and 95% confidence interval (95% CI) of breast cancer by adherence to the serum vitamin-mineral profiles and single biomarkers (*n* = 129).

Serum ‘Vitamin-Mineral’ Profiles/Single Biomarkers	Tertiles/Levels	Sample Size	Breast Cancer						
			Percentage (%)	Unadjusted Model 1		Model 2		Model 3	
				ORs	95% CI	ORs	95% CI	ORs	95% CI
‘Folate-Cobalamin-Vitamin D’	bottom (ref.)	43	40	1.00 (ref.)		1.00 (ref.)		1.00 (ref.)	
	middle	43	29	0.54	0.22; 1.30	0.64	0.19; 2.15	0.67	0.18; 2.45
	upper	43	31	0.62	0.26; 1.49	1.27	0.47; 3.42	1.30	0.46; 3.66
‘Iron-Calcium’	bottom (ref.)	43	46	1.00 (ref.)		1.00 (ref.)		1.00 (ref.)	
	middle	42	29	0.46	0.19; 1.11	0.26 *	0.07; 0.94	0.31	0.08; 1.21
	upper	44	25	0.34 *	0.14; 0.84	0.24 *	0.06; 0.99	0.12 *	0.02; 0.88
‘Magnesium’	bottom (ref.)	43	47	1.00 (ref.)		1.00 (ref.)		1.00 (ref.)	
	middle	42	35	0.62	0.26; 1.48	0.56	0.16; 1.90	0.73	0.20; 2.68
	upper	44	18	0.23 **	0.09; 0.61	0.21 *	0.05; 0.84	0.26	0.05; 1.25
Folate (ng/mL)	<10.5 (ref.)	64	39	1.00 (ref.)		1.00 (ref.)		1.00 (ref.)	
	≥10.5	65	61	2.07	0.97; 4.31	2.02	0.91; 4.23	2.01	0.90; 4.27
Cobalamin (pg/mL)	<341.65 (ref.)	64	59	1.00 (ref.)		1.00 (ref.)		1.00 (ref.)	
	≥341.65	65	41	0.55	0.27; 1.14	0.72	0.29; 1.83	0.66	0.24; 1.79
25(OH) vitamin D (ng/mL)	<24.6 (ref.)	64	66	1.00 (ref.)		1.00 (ref.)		1.00 (ref.)	
	≥24.6	65	34	0.34 **	0.16; 0.71	0.28 *	0.10; 0.78	0.33 *	0.11; 0.97
Iron (μg/dL)	<103.0 (ref.)	64	62	1.00 (ref.)		1.00 (ref.)		1.00 (ref.)	
	≥103.0	65	38	0.44 *	0.21; 0.92	0.72	0.28; 1.82	0.68	0.24; 1.92
Calcium (mg/dL)	<9.6 (ref.)	51	54	1.00 (ref.)		1.00 (ref.)		1.00 (ref.)	
	≥9.6	78	46	0.38 **	0.18; 0.79	0.33 *	0.12; 0.87	0.32 *	0.11; 0.91
Magnesium (mg/dL)	<2.1 (ref.)	47	44	1.00 (ref.)		1.00 (ref.)		1.00 (ref.)	
	≥2.1	82	56	0.60	0.29; 1.25	0.54	0.21; 1.38	0.63	0.23; 1.72

ref.—referent, the reference categories were the control sample and the bottom tertile of serum ‘Vitamin-Mineral’ profiles or lowest level of each single serum biomarkers; Model 2—age (years), BMI (kg/m^2^), socioeconomic status (low, average, high), overall physical activity (low, moderate, high), smoking status (non-smoker, smoker), age at menarche (<12, 12–14.9, ≥15 years), number of full-term pregnancies (0, 1–2, ≥3), oral contraceptive use (no, yes), hormone-replacement therapy use (no, yes), family history of breast cancer in first- or second-degree relative (no, I don’t know, yes), vitamin/mineral supplements use (no, yes) and molecular of breast cancer subtypes (triple negative, ER-, PR-, HER2+ subtype, luminal A, luminal B) adjusted model; Model 3—model was adjusted for the same variables included in model 2 plus PCA-driven DP scores (fully-adjusted model); 95% CI—95% confidence interval; *p*-value—the level of significance verified with Wald’s test; * *p* < 0.05, ** *p* < 0.01.

## Supplementary Materials

The following are available online at https://www.mdpi.com/2072-6643/11/9/2244/s1, Table S1: Cancer-control sample and its sub-sample characteristics (%), Table S2: Cancer sample and its sub-sample characteristics (%), Table S3: Control sample and its sub-sample characteristics (%), Table S4: The cancer-control sub-sample characteristics by the serum vitamin-mineral profiles (%), Table S5: Serum vitamins and mineral concentrations by the serum vitamin-mineral profiles (%) among postmenopausal women (*n* = 129), Table S6: Serum vitamins and minerals concentration by the serum vitamin-mineral profiles (%) among postmenopausal women (*n* = 129). Figure S1: Histograms of serum 25(OH) vitamin D and calcium concentrations among control and cancer sub-samples.
