# Supplementary material for: Serum ‘Vitamin-Mineral’ Profiles: Associations with Postmenopausal Breast Cancer Risk Including Dietary Patterns and Supplementation. A Case-Control Study
Source: Nutrients. 2019 Sep 18;11(9):2244. doi: 10.3390/nu11092244 (PMC6770708; doi:10.3390/nu11092244)
Supplement: Supplementary file 1 [file nutrients-11-02244-s001.pdf]

Table S1. Description of 21 food groups aggregated: data based on the FFQ-6 questionnaire [48].

No	Food groups	Food groups description
1	Sugar, honey and sweets	Sugar added to beverages, such as tea, coffee, etc.;<br>Honey added to dishes and added to beverages;<br>Chocolates, chocolate sweets and chocolate bars, sugar confectionery (boiled sweets, hard caramels, jellied sweets, fudge, etc.), baked confectionery (biscuits, cream cakes, fruit cakes, sponge cakes, cheesecakes, doughnuts, poppy-seed cakes, muffins, croissants, etc.), ice-creams and custard.
2	Milk and milk beverages – natural and cheese curds	Milk and natural milk beverages (yoghurt, kefir, buttermilk), porridge, etc.<br>Cheese curd, natural cottage cheese, soft cheese, mozzarella, cottage cheese with herbs, etc.
3	Milk beverages – sweetened and flavoured cheese curds	Fruit yoghurts, yoghurts with chocolate flakes, flavoured buttermilk, hot chocolate, etc.<br>Flavoured curds (with fruit, chocolate, vanilla), etc.
4	Cheese	Hard cheese, blue cheese, processed cheese, cheese spreads, etc.
5	Eggs and egg dishes	Scrambled eggs, omelette, egg salad, cooked eggs.
6	Breakfast cereals	Muesli, cornflakes, other cereals - sweetened or unsweetened, etc.
7	Wholemeal cereals and coarse groats	Wholemeal wheat or rye bread, seeded loafs, pumpernickel, wholemeal cracker bread, etc.<br>Buckwheat groats, barley, brown rice, wholemeal pasta, etc.
8	Refined cereals and fine groats	White bread, rye, wheat-rye bread, toast bread, white bread rolls, brioche, bagels, etc.<br>Semolina, milled barley, pasta, white rice, rice flakes, etc.
9	Animal fats	Butter;<br>Lard, pork fat, etc.<br>Cream (single, double, sour, used as an ingredient or added to beverages).
10	Vegetable based oil	Vegetable oils;<br>Olive oil.
11	Other fats	Margarine for baking, frying, spreading, mayonnaise and salad dressings.
12	Fruits	All kinds of fruits.
13	Vegetables	All kinds of vegetables (potatoes not included).
14	Potatoes	Boiled, baked, French fries, potato rosti, gnocchi, etc.
15	Nuts and seeds	Peanuts, hazelnuts, walnuts, cashews, coconuts, chestnuts, peanut butter, chocolate-nut spread, etc.<br>Pumpkin seeds, sesame seeds, sunflower seeds, wheat germs, wheat bran, etc.
16	Legumes	Fresh and tinned legumes (corn, green peas, green beans, etc.);<br>Dry and processed pulses (beans (fava, butter kidney, broad, French, green), soya, peas, chickpea and processed pulses (baked beans, hummus, other bread spreads)).
17	White meat	Poultry and rabbit.
18	Red and processed meats	Red meat (pork, beef, veal, etc.)<br>Venison (wild boar, venison, quail, mallard, hare, etc.)<br>Sausages, bacon, reconstituted meat (sausages, meat loaf, hot-dogs, smoked sausages, bacon, etc.)<br>High quality cured meats (ham, poultry and pork-beef good quality cold meats, etc.)<br>Offal products (liver, blood sausage, sweetbread, liver pate, etc.).
19	Fish	Lean fish (pollock, cod, perch, hake, carp to 1 kg, tuna, panga, trout, etc.)<br>Oily fish (salmon, sardines, herring, mackerel, eel, large carp, etc.).
20	Fruit, vegetable or vegetable-fruit juices	Fruit juices and nectars (mixed fruit juice, orange, grapefruit, apple, pear, grape, blackcurrant, cherry juice)<br>Vegetable and vegetable-fruit juices (mixed vegetable juice, tomato, carrot and carrot-fruit juice).
21	Sweetened beverages and energy drinks	All kinds of sweetened beverages.<br>All kinds of energy drinks.

Table S2. Cancer-control sample and its sub-sample characteristics (%).

Variable	Cancer-Control sample	Cancer-Control sub-sample	*p*-Value
Sample Size	420	129	
Age (years#)	59.9 (8.6)	61.9 (8.2)	0.0199
BMI (kg/m2#)	27.9 (5.0)	27.9 (5.1)	ns
Socioeconomic status (SES Index#)	9.9 (2.1)	9.9 (2.3)	ns
low	41.0	40.8	ns
average	36.7	35.4	ns
high	22.4	23.8	ns
Overall physical activity			
low	52.9	56.2	ns
moderate	44.0	41.5	ns
high	3.1	2.3	ns
Smoking status (smoker$)	53.1	46.2	ns
Abuse of alcohol	4.0	0.0	ns
Age at menarche (years)			
<12	12.1	7.7	ns
12-14.9	63.3	70.0	ns
≥15	24.5	22.3	ns
Menopausal status			
pre-menopausal	14.8	0.0	0.0001
post-menopausal	85.2	100.0	ns
Number of full-term pregnancies			
0	12.1	10.8	ns
1-2	61.7	62.3	ns
≥3	26.2	26.9	ns
Oral contraceptive use (ever)	20.2	23.8	ns
Hormone-replacement therapy use (ever)	16.7	22.3	ns
Family history of BC&	19.3	23.8	ns
Vitamin/mineral supplements use	38.6	53.8	0.0022
Dietary patterns score (points)#			
'Non-Healthy'	3.5 (1.8)	2.9 (1.7)	0.0009
'Prudent'	3.4 (1.2)	3.4 (1.3)	ns
'Margarine and Sweetened Dairy'	0.1 (1.0)	-0.1 (0.9)	ns

BMI – body mass index; SES – socioeconomic status; BC – breast cancer; $current and/or former smoker; &in first- or second-degree relative; 'Polish-aMED' – 'Polish-adapted Mediterranean Diet' (range of points: 0-8); % – sample percentage; #mean and standard deviation (SD); *p*-value – level of significance verified with chi^2 test (categorical variables) or Kruskal-Wallis' test (continuous variables); $p < 0.05$; ns – statistically insignificant.

Table S3. Cancer sample and its sub-sample characteristics (%).

Variable	Cancer sample	Cancer sub-sample	*p*-Value
Sample Size	190	47	
Age (years#)	60.9 (9.7)	62.2 (10.4)	ns
BMI (kg/m2#)	28.3 (4.8)	28.8 (5.1)	ns
Socioeconomic status (SES Index#)	9.3 (2.1)	8.4 (1.8)	0.0074
low	53.2	69.4	0.0449
average	33.2	28.6	ns
high	13.7	2.0	0.0081
Overall physical activity			
low	67.9	71.4	ns
moderate	30.5	26.5	ns
high	1.6	2.0	ns
Smoking status (smoker$)	57.9	55.1	ns
Abuse of alcohol	7.4	0.0	ns
Age at menarche (years)			
<12	16.3	14.3	ns
12-14.9	63.2	67.4	ns
≥15	20.5	18.4	ns
Menopausal status			
pre-menopausal	15.3	0.0	0.0003
post-menopausal	84.7	100.0	0.0003
Number of full-term pregnancies			
0	7.9	4.1	ns
1-2	61.6	55.1	ns
≥3	30.5	40.8	ns
Oral contraceptive use (ever)	17.9	18.4	ns
Hormone-replacement therapy use (ever)	15.3	20.4	ns
Family history of BC&	24.7	32.7	ns

Vitamin/mineral supplements use	31.1	42.9	ns
Molecular subtypes of BC^			
Triple negative (ER-, PR-, HER2-)	12.1	10.2	ns
ER-, PR-, HER2+ subtype	3.6	0.0	ns
Luminal A (ER+ and or PR+, HER2-)	70.0	73.5	ns
Luminal B (ER+ and or PR+, HER2+)	14.3	16.3	ns
Dietary patterns score (points)#			
'Non-Healthy'	4.1 (1.9)	3.6 (1.7)	ns
'Prudent'	3.3 (1.2)	3.1 (1.5)	ns
'Margarine and Sweetened Dairy'	0.2 (1.0)	0.1 (1.0)	ns

BMI – body mass index; SES – socioeconomic status; BC – breast cancer; $current and/or former smoker; &in first- or second-degree relative; 'Polish-aMED' – 'Polish-adapted Mediterranean Diet' (range of points: 0-8); ^data for n=140; ER – oestrogen receptor status of tumour; PR – progesterone receptor status of tumour; HER2 – human epidermal growth factor receptor 2; % – sample percentage; #mean and standard deviation (SD); *p*-value – level of significance verified with chi^2 test (categorical variables) or Kruskal-Wallis' test (continuous variables); $p < 0.05$; ns – statistically insignificant.

Table S4. Control sample and its sub-sample characteristics (%).

Variable	Control sample	Control sub-sample	*p*-Value
Sample Size	230	82	
Age (years#)	59.1 (7.4)	61.7 (6.7)	0.0055
BMI (kg/m^2#)	27.6 (5.0)	27.3 (5.1)	ns
Socioeconomic status (SES Index#)	10.4 (2.0)	10.8 (2.1)	ns
low	30.9	23.5	ns
average	39.6	39.5	ns
high	29.6	37.0	ns
Overall physical activity			
low	40.4	46.9	ns
moderate	55.2	50.6	ns
high	4.3	2.5	ns
Smoking status (smoker$)	49.1	40.7	ns
Abuse of alcohol	1.3	0.0	ns
Age at menarche (years)			
<12	8.7	3.7	ns
12-14.9	63.5	71.6	ns
≥15	27.8	24.7	ns
Menopausal status			
pre-menopausal	14.3	0.0	0.0003
post-menopausal	85.7	100.0	0.0003
Number of full-term pregnancies			
0	15.7	14.8	ns
1-2	61.7	66.7	ns
≥3	22.6	18.5	ns
Oral contraceptive use (ever)	22.2	27.2	ns
Hormone-replacement therapy use (ever)	17.8	23.5	ns
Family history of BC&	14.8	18.5	ns
Vitamin/mineral supplements use	44.8	60.5	0.0146
Dietary patterns score (points)#			
'Non-Healthy'	3.1 (1.6)	2.5 (1.5)	0.0033
'Prudent'	3.5 (1.3)	3.5 (1.1)	ns
'Margarine and Sweetened Dairy'	0.1 (1.0)	-0.3 (0.8)	0.0012

BMI – body mass index; SES – socioeconomic status; BC – breast cancer; $current and/or former smoker; &in first- or second-degree relative; 'Polish-aMED' – 'Polish-adapted Mediterranean Diet' (range of points: 0-8); % – sample percentage; #mean and standard deviation (SD); *p*-value – level of significance verified with chi^2 test (categorical variables) or Kruskal-Wallis' test (continuous variables); $p < 0.05$; ns – statistically insignificant.

Table S5. The cancer-control sub-sample characteristics by the serum vitamin-mineral profiles (%).

Variable	Serum vitamin-mineral profiles											
	'Folate-Cobalamin-Vitamin D' tertiles				'Iron-Calcium' tertiles				'Magnesium' tertiles			
	bottom	middle	upper	*p*-Value	bottom	middle	upper	*p*-Value	bottom	middle	upper	*p*-Value
Sample size (n)	43	43	43		43	42	44		43	42	44	
Age (years#)	62.7 (9.4)	61.2 (7.4)	61.8 (7.9)	ns	61.2 (9.7)	61.5 (6.3)	62.9 (8.4)	ns	61.2 (9.0)	61.8 (8.0)	62.6 (7.8)	ns
BMI ($kg/m^{2\#}$)	27.8 (5.3)	28.5 (5.3)	27.2 (4.8)	ns	27.5 (5.4)	28.4 (4.9)	27.7 (5.1)	ns	28.2 (5.5)	28.3 (4.9)	27.1 (4.9)	ns
Socioeconomic status (SES Index#)	9.5 (2.4)	9.6 (2.3)	10.6 (2.1)	ns	9.5 (2.4)	9.9 (2.3)	10.4 (2.2)	ns	9.5 (2.4)	9.8 (2.2)	10.5 (2.2)	ns
low	46.5	45.5	30.2		50.0	40.5	31.8		50.0	45.2	27.3	
average	37.2	31.8	37.2	ns	27.3	38.1	40.9	ns	29.5	33.3	43.2	ns
high	16.3	22.7	32.6		22.7	21.4	27.3		20.5	21.4	29.5	
Overall physical activity												
low	60.5	61.4	46.5		59.1	61.9	47.7		50.0	71.4	47.7	
moderate	39.5	36.4	48.8	ns	38.6	38.1	47.7	ns	45.5	28.6	50.0	ns
high	0.0	2.3	4.7		2.3	0.0	4.5		4.5	0.0	2.3	
Smoking status (smoker$)	48.8	50.0	39.5	ns	45.5	54.8	38.6	ns	50.0	47.6	40.9	ns
Abuse of alcohol	0.0	0.0	0.0	ns	0.0	0.0	0.0	ns	0.0	0.0	0.0	ns
Age at menarche (years)												
<12	9.3	6.8	7.0		9.1	9.5	4.5		6.8	11.9	4.5	
12-14.9	69.8	68.2	72.1	ns	72.7	64.3	72.7	ns	72.7	71.4	65.9	ns
≥15	20.9	25.0	20.9		18.2	26.2	22.7		20.5	16.7	29.5	
Number of full-term pregnancies												
0	4.7	6.8	20.9		4.5	11.9	15.9		11.4	9.5	11.4	
1-2	55.8	77.3	53.5	0.0103	61.4	64.3	61.4	ns	52.3	66.7	68.2	ns
≥3	39.5	15.9	25.6		34.1	23.8	22.7		36.4	23.8	20.5	
Oral contraceptive use (ever)	25.6	29.5	16.3	ns	29.5	23.8	18.2	ns	20.5	28.6	22.7	ns
Hormone-replacement therapy use (ever)	14.0	20.5	32.6	ns	6.8	31.0	29.5	0.0099	25.0	16.7	25.0	ns
Family history of BC&	23.3	25.0	23.3	ns	25.0	23.8	22.7	ns	25.0	28.6	18.2	ns
Vitamin/mineral supplements use	32.6	52.3	76.7	0.0002	52.3	42.9	65.9	ns	47.7	47.6	65.9	ns

BMI – body mass index; SES – socioeconomic status; BC – breast cancer; $current and/or former smoker; &in first- or second-degree relative; % – sample percentage; #mean and standard deviation (SD); *p*-value – level of significance verified with chi^2 test (categorical variables) or Kruskal-Wallis' test (continuous variables); $p < 0.05$; ns – statistically insignificant.

Table S6. Serum vitamins and minerals concentration by the serum vitamin-mineral profiles (%) among postmenopausal women (n=129).

**Serum vitamins/minerals concentration**	**Serum vitamin-mineral profiles**											
	**'Folate-Cobalamin-Vitamin D' tertiles**				**'Iron-Calcium' tertiles**				**'Magnesium' tertiles**			
	**bottom**	**middle**	**upper**	***p*-Value**	**bottom**	**middle**	**upper**	***p*-Value**	**bottom**	**middle**	**upper**	***p*-Value**
Sample Size (n)	43	43	43		43	42	44		43	42	44	
Folate (ng/mL)#	7.3 (3.5)	10.7 (4.0)	17.5 (9.2)	<0.0001	14.0 (9.5)	10.3 (6.1)	11.1 (5.6)	ns	13.3 (9.8)	10.8 (6.2)	11.3 (5.4)	ns
5th; 95th percentile	2.8; 12.4	5.2; 18.2	8.5; 32.8		4.3; 32.5	3.5; 23.0	3.7; 18.0		4.7; 32.8	3.6; 20.0	4.3; 23.0	
≥10.5	18.6	47.7	83.7	<0.0001	59.1	35.7	54.5	ns	52.3	47.6	50.0	ns
Cobalamin (pg/mL) #	243.8 (70.1)	361.1 (80.6)	502.8 (183.0)	<0.0001	341.4 (201.5)	364.9 (136.0)	401.1 (132.4)	0.0127	397.0 (191.5)	331.9 (123.4)	377.0 (55.7)	ns
5th; 95th percentile	147.6; 385.5	247.3; 509.2	285.1; 762.2		147.6; 762.2	208.7; 605.9	220.7; 659.3		161.3; 575.4	186.4; 604.7	191.7; 695.8	
≥341.65	9.3	59.1	81.4	<0.0001	34.1	47.6	68.2	0.0056	52.3	40.5	56.8	ns
25(OH) vitamin D (ng/mL)#	21.8 (8.6)	22.8 (9.6)	35.4 (17.0)	<0.0001	26.8 (16.8)	25.0 (10.5)	28.1 (13.0)	ns	23.0 (11.7)	23.6 (9.5)	33.1 (16.5)	0.0003
5th; 95th percentile	8.8; 36.7	9.8; 37.7	15.0; 60.8		9.8; 48.9	10.7; 42.4	8.9; 46.5		8.8; 46.5	10.8; 40.5	14.5; 52.8	
≥24.6	39.5	36.4	74.4	0.0005	47.7	47.6	54.5	ns	36.4	42.9	70.5	0.0032
Iron (μg/dL) #	98.8 (29.1)	104.3 (30.1)	109.9 (29.8)	ns	78.1 (17.7)	102.6 (14.1)	132.1 (25.7)	<0.0001	112.5 (32.0)	98.0 (28.6)	102.2 (27.4)	ns
5th; 95th percentile	58.7; 143.0	62.6; 154.1	59.7; 161.6		55.0; 103.1	79.7; 124.0	93.7; 169.4		72.5; 169.4	55.8; 148.8	71.6; 149.4	
≥103.0	41.9	50.0	60.5	ns	9.1	57.1	86.4	<0.0001	61.4	42.9	47.7	ns
Calcium (mg/dL) #	9.5 (0.4)	9.7 (0.4)	9.6 (0.6)	0.0381	9.2 (0.5)	9.6 (0.2)	9.9 (0.3)	<0.0001	9.5 (0.6)	9.5 (0.4)	9.7 (0.3)	0.0041
5th; 95th percentile	8.8; 9.9	8.9; 10.0	8.7; 10.3		8.6; 9.8	9.3; 9.9	9.4; 10.5		8.7; 10.1	8.6; 9.9	9.3; 10.2	
≥9.6	48.8	65.9	67.4	ns	27.3	66.7	88.6	<0.0001	56.8	42.9	81.8	0.0009
Magnesium (mg/dL) #	2.1 (0.1)	2.1 (0.1)	2.1 (0.1)	ns	2.1 (0.1)	2.1 (0.1)	2.1 (0.1)	ns	2.0 (0.1)	2.1 (0.1)	2.2 (0.1)	<0.0001
5th; 95th percentile	1.9; 2.3	1.8; 2.4	1.9; 2.3		1.9; 2.3	1.9; 2.3	1.8; 2.3		1.8; 2.1	2.0; 2.2	2.1; 2.4	
≥ 2.1	65.1	70.5	53.5	ns	61.4	64.3	63.6	ns	11.4	78.6	100.0	<0.0001

% – sample percentage; #mean and standard deviation (SD); *p*-value – level of significance verified with by chi^2 test (categorical variables) or Student's t-test (for continuous variables, log-transformed serum biomarkers concentration); $p < 0.05$; ns – statistically insignificant.

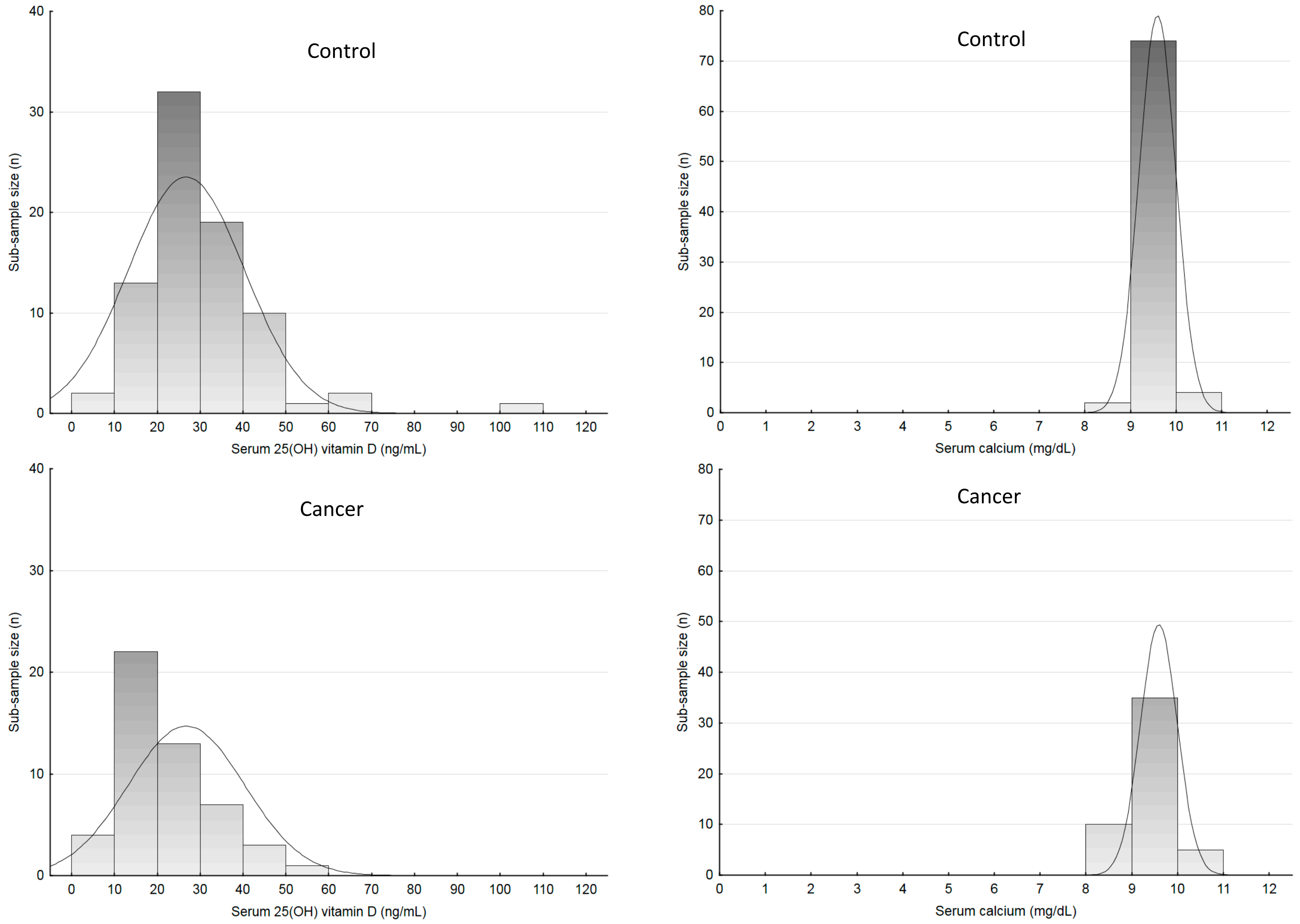

Figure S1. Histograms of serum 25(OH) vitamin D and calcium concentrations among control and cancer sub-samples.
